# Malignancy Ratio in Pediatric Patients with Hereditary Multiple Exostoses: True Association or Reporting Bias?

**DOI:** 10.3390/pediatric17060132

**Published:** 2025-12-03

**Authors:** Francesco Fabrizio Comisi, Andrea Maria Comisi, Elena Esposito, Salvatore Savasta

**Affiliations:** 1 Pediatric Clinic and Rare Diseases, Microcitemico Hospital “A. Cao”, University of Cagliari, 09124 Cagliari, Italy; 2 Department of Clinical and Experimental Medicine, University of Catania, 95131 Catania, Italy; 3 Pediatric Clinic and Rare Diseases, Microcitemico Hospital “A. Cao”, Department of Medical Sciences and Public Health, University of Cagliari, 09124 Cagliari, Italy

**Keywords:** Hereditary Multiple Exostoses, Hereditary Multiple Osteochondromas, malignancy, cancer, leukemia, children

## Abstract

Background: Hereditary Multiple Exostoses (HME) is a rare autosomal dominant skeletal disorder resulting from loss-of-function variants in the *EXT1*, *EXT2*, or *EXT3* genes. While malignant transformation into chondrosarcoma is well documented, the incidence and characterization of non-skeletal malignancies in HME remain poorly defined. Objective: We aimed to comprehensively review the literature for reported cases of non-skeletal malignancies in individuals with HME and evaluate a potential association with hematologic cancers, particularly in the pediatric population. Methods: An extensive literature search was conducted in the PubMed database up to August 2025 using search terms related to HME and malignancy. Eligible reports included case descriptions of non-skeletal cancers occurring in patients with confirmed or suspected HME. Extracted data included patient age, sex, cancer type, and available genetic or molecular findings. Results: Thirteen cases of non-skeletal malignancies associated with HME were identified. Fewer than half underwent molecular genetic testing. Six cases occurred in pediatric patients, four of which involved hematologic malignancies, three leukemias and one Burkitt lymphoma. In adults, malignancies affected a range of organ systems, including respiratory, gastrointestinal, nervous, and endocrine. A marked male predominance was observed (11 males vs. 2 females). Conclusions: Although a definitive causal relationship cannot be established, hematologic malignancies in pediatric HME patients appear to be disproportionately represented among reported cases. This finding highlights the need for further investigation through large-scale, population-based studies incorporating both clinical and genetic data.

## 1. Introduction

HME is a rare autosomal dominant skeletal disorder caused by loss-of-function variants in the *EXT1* (8q24), *EXT2* (11p11–p13), or *EXT3* (19p) genes [1]. It is characterized by the formation of multiple benign, cartilage-capped bony outgrowths (osteochondromas) that arise from the perichondrium adjacent to regions of actively growing cartilage. The estimated prevalence is approximately 1 in 50,000 individuals [2,3]. Also referred to as Hereditary Multiple Osteochondromas (HMO) or Hereditary Deforming Dyschondroplasia, HME exhibits nearly complete penetrance, particularly in males. More than half of affected individuals inherit the condition from a parent, most often the father [4,5,6,7,8,9,10]. Typically, asymptomatic males do not transmit the pathogenic variant, whereas asymptomatic females may act as silent carriers [11]. HME accounts for only 5–10% of all cases of exostosis, with most lesions being solitary. Diagnosis is usually established before the age of 10 and occurs more frequently in males, with a male-to-female ratio of approximately 1.5:1 [5,6]. Osteochondromas most commonly affect the metaphyseal regions of long bones but may also involve the scapulae, ribs, pelvis, and vertebrae. In contrast, the skull, carpal, and tarsal bones are generally spared [6,12]. Clinical severity varies depending on the number, morphology, and anatomical distribution of lesions, as well as the risk of malignant transformation into chondrosarcoma [2,13,14,15]. Osteochondromas can impair longitudinal bone growth, leading to limb-length discrepancies, skeletal deformities, and joint complications, particularly at the hip, such as coxa valga, acetabular dysplasia, femoroacetabular impingement, and early-onset osteoarthritis [12,16]. Additional complications may include chronic pain, nerve entrapment, restricted mobility, and inflammatory symptoms. Short stature is commonly observed, especially among individuals harboring pathogenic *EXT1* variants [12]. Management is primarily surgical and includes excision of symptomatic lesions, limb-lengthening procedures, and corrective osteotomies. Surgical intervention is generally reserved for patients with significant functional impairment or when malignant transformation is suspected [17].

## 2. Materials and Methods

**Figure 1:**
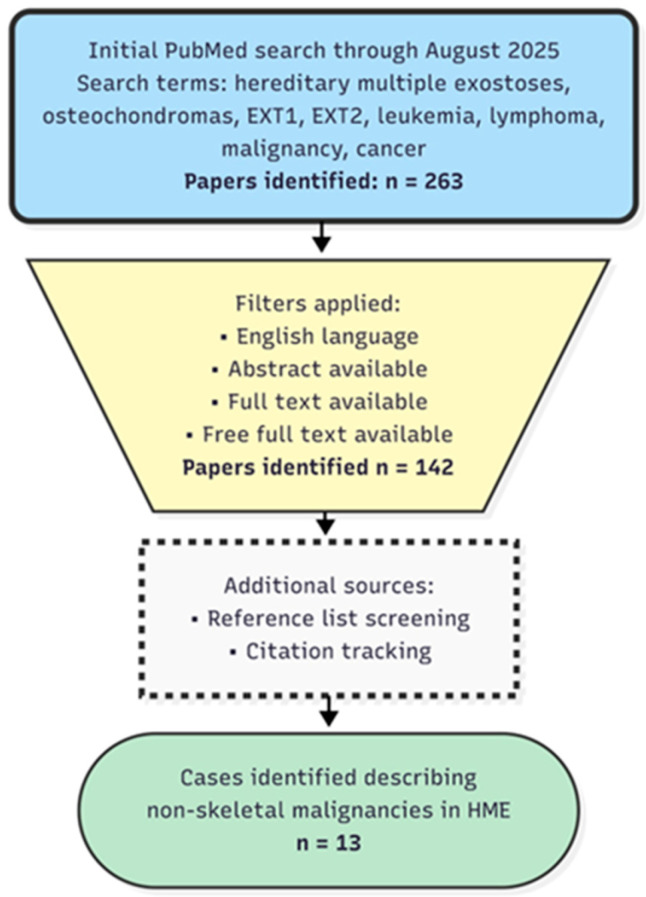
**Literature search and case identification strategy.** The initial PubMed search yielded 263 papers. After applying language and abstract availability filters, 142 papers were screened. Additional cases were identified through manual reference list screening and citation tracking of key publications.

An extensive literature search was conducted using the PubMed database to identify reports of malignancies occurring in individuals with Hereditary Multiple Exostoses. The search, carried out through August 2025, employed combinations of the following terms: “hereditary multiple exostoses,” “osteochondromas,” “*EXT1*,” “*EXT2*,” “leukemia,” “lymphoma,” “malignancy,” and “cancer.” No language restrictions were applied. Case reports and case series were included if they described the coexistence of HME and a malignancy other than chondrosarcoma. Reports focusing exclusively on skeletal malignancies were excluded from the analysis of non-skeletal tumors. A total of 13 cases were identified and included in the final analysis (Figure 1). When available, the following data were extracted: patient age and sex, type and anatomical site of malignancy, and findings from any genetic or molecular analyses. Reported malignancies were categorized into five anatomical systems: hematopoietic, respiratory, gastrointestinal, nervous, and endocrine. Data were analyzed descriptively, with particular attention to age distribution and sex differences.

## 3. Results

### 3.1. Skeletal Malignancies

The association between HME and skeletal malignancies is well established. Malignant transformation of osteochondromas occurs in approximately 4–10% of affected individuals, with peak incidence in the third decade of life [18,19,20]. Histologically, approximately 94% of these malignant lesions are classified as chondrosarcomas [17,21,22], which are typically low-grade tumors associated with a favorable prognosis; reported long-term survival rates range from 70% to 90% [18]. Less frequent histological subtypes include osteosarcoma, fibrosarcoma, and malignant fibrous histiocytoma [2]. Notably, carriers of *EXT1* mutations appear to have a higher risk of malignant transformation compared to those with *EXT2* variants [17].

### 3.2. Hematologic Malignancies

To date, three cases of leukemia have been reported in association with HME. The first, described in Turkey, involved an 8-year-old girl diagnosed with acute myeloid leukemia; no genetic testing was performed [23]. A second report, from Japan, described a 14-month-old boy with pre-B acute lymphoblastic leukemia and an *EXT1* deletion. The authors proposed a potential leukemogenic role for the gene alteration [24]. The third case, reported in Italy, involved a 14-year-old girl with pre-B acute lymphoblastic leukemia and an *EXT2* mutation. It was suggested that disruption of heparan sulfate biosynthesis due to *EXT2* dysfunction may have contributed to malignant transformation [25]. Two additional cases of lymphoma have also been reported. One involved a 52-year-old man from Germany with high-grade non-Hodgkin lymphoma of the bone [26], and the other, a 10-year-old boy from the United States diagnosed with abdominal Burkitt lymphoma [27]. Neither case included molecular genetic analysis.

### 3.3. Other Malignancies

Two cases of lung cancer have been reported in individuals with Hereditary Multiple Exostoses. The first, published in Russia in 1981, involved a 56-year-old man with a history of heavy smoking and a family history of pulmonary disease. Histological examination identified a squamous cell carcinoma, likely attributable to tobacco exposure [28,29]. The second case, reported in China in 2020, concerned a 33-year-old man with no history of smoking or alcohol use who was diagnosed with primary lung adenocarcinoma; a metastatic origin was excluded [30]. In both cases, no *EXT* gene analysis was performed. Two cases of intestinal malignancy were also identified. In 2009, Italian researchers described a 31-year-old man with juvenile-onset colon carcinoma harboring a missense variant in *EXTL3* [31]. The second case, from Ghana in 2023, involved a 12-year-old boy diagnosed with colorectal cancer; no genetic evaluation was reported [32]. Sporadic cases of central nervous system tumors have likewise been reported. A 15-year-old boy from the United States was diagnosed with cerebellar astrocytoma [33], and an 18-year-old male in China was found to have an intracranial atypical teratoid/rhabdoid tumor [34]. Neither case included molecular genetic testing. One case of papillary thyroid carcinoma was described in a 36-year-old man from the Netherlands; no *EXT1* or *EXT2* mutations were identified [2]. Finally, in 2015, a German group reported a case of multiple endocrine neoplasia type 1 (MEN1) in a 47-year-old man carrying both a novel *EXT1* mutation and a pathogenic variant in the *MEN1* gene [35].

## 4. Discussion

Our comprehensive literature review identified 13 reported cases of non-skeletal malignancies in individuals with HME. Fewer than half (5/13) underwent genetic or molecular analysis (Table 1).

**Figure 2:**
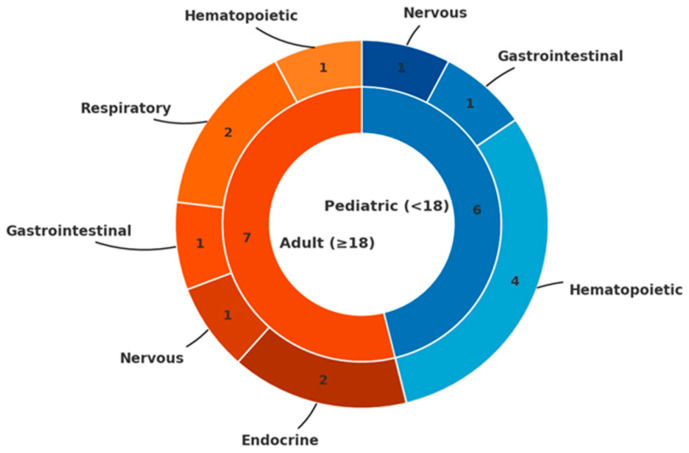
**Distribution of non-skeletal malignancies in Hereditary Multiple Exostoses (HME) by age group and organ system.** Concentric donut chart: inner ring shows age group (Pediatric < 18 years n = 6, Adult ≥ 18 years n = 7); outer ring shows organ system categories (hematopoietic, respiratory, gastrointestinal, nervous, endocrine) with casa counts per age group. Colors: blue = pediatric, orange = adult, Total n = 13.

The majority of cases occurred in males (11 males vs. 2 females), yielding a male-to-female ratio of 5.5:1, substantially higher than the 1.2:1 ratio reported in general cancer epidemiology [36,37]. The distribution of pediatric versus adult cases (6 vs. 7) contrasts with the expected rarity of childhood cancers, which account for only 1.2% of all malignancies [38]. Among the six pediatric cases identified, four involved hematologic malignancies (67%), yielding an observed-to-expected ratio of approximately 185, far from the baseline prevalence of hematologic cancers in general pediatric population, which account for 25–30% of childhood malignancies [38] (Figure 2). This apparent overrepresentation, however, must be interpreted with caution given the small sample size and the substantial influence of publication bias in such rare co-occurrences. The marked male predominance similarly deviates from expected cancer epidemiology patterns, further suggesting selective reporting rather than a true biological association. The observed predominance of pediatric hematologic malignancies warrants cautious interpretation considering potential reporting bias. Malignancy in children is rare, representing only 1.2% of all cancers, and its co-occurrence with HME is likely to prompt case reporting due to perceived novelty. Common adult malignancies in HME patients may lack sufficient clinical interest for publication, leading to systematic underrepresentation. Hematologic malignancies in children are typically managed in specialized centers where genetic workup is more routine, increasing the likelihood of identifying HME as a comorbidity. The mechanistic plausibility of EXT gene involvement in hematopoietic pathways may create confirmation bias favoring publication of leukemia cases. The marked male predominance (11:2) similarly exceeds expected patterns and may reflect preferential reporting rather than genuine sex-specific risk. Without systematic surveillance data or population-based registries, distinguishing true association from publication artifacts remains impossible. Large-scale cohort studies are needed to clarify whether HME confers increased risk for specific non-skeletal malignancies.

## 5. Study Limitations

This review has several important limitations. The findings are hypothesis-generating, based on only 13 case reports with insufficient statistical power to establish causal associations. The absence of denominator data prevents calculation of incidence rates or relative risks, leaving uncertain whether observed cases represent genuine increased risk or sporadic co-occurrence. Publication bias represents a significant concern, as unusual associations in pediatric populations are preferentially reported, potentially inflating the apparent frequency of hematologic malignancies in children with HME, while common adult cancers co-occurring with HME may be underreported. The observed male predominance may similarly reflect reporting preferences rather than biological predisposition.

Inconsistent genetic testing (5/13 cases) limits genotype-phenotype correlation, and heterogeneity in clinical detail and reporting quality further complicates interpretation since other skeletal dysplasias or syndromes might have been erroneously reported as HME. The apparent overrepresentation of pediatric hematologic malignancies should therefore be considered a preliminary observation requiring validation through large-scale, population-based studies with appropriate controls.

## 6. Conclusions

Based on the currently available evidence, a definitive association between HME and non-skeletal malignancies cannot be established. This review identified an apparent overrepresentation of pediatric hematologic malignancies (4 of 6 pediatric cases), but the small sample size, absence of denominator data, and substantial risk of publication bias limit causal inference. The findings are hypothesis-generating and require validation through large-scale, population-based cohort studies with appropriate controls. Although no screening recommendations can currently be made, clinicians should maintain awareness of potential hematologic malignancies in pediatric patients with HME presenting with unexplained constitutional symptoms. Future research incorporating clinical, genetic, and epidemiological data is essential to determine whether HME confers genuine increased risk for specific non-skeletal cancers.

## Figures and Tables

**Table 1:** Non-skeletal malignancies reported in individuals with Hereditary Multiple Exostoses.

System	Pt	Malignancy	Age	Sex	Genetic Anomaly
**Hematopoietic**	1	AML	8 y	F	Not available
	2	Pre-B ALL	14 m	M	*EXT1* deletion
	3	Pre-B ALL	14 y	F	*EXT2* mutation
	4	High grade NHL	53 y	M	Not available
	5	BL (abdomen)	10 y	M	Not available
**Respiratory**	6	Lung SCC (uncertain correlation)	56 y	M	Not available
	7	Lung adenocarcinoma	33 y	M	Not available
**Gastrointestinal**	8	Colon carcinoma	31 y	M	*EXTL3* variant
	9	Colorectal carcinoma	12 y	M	Not available
**Nervous**	10	Cerebellar astrocytoma	15 y	M	Not available
	11	Atypical teratoid/rhabdoid tumor	18 y	M	Not available
**Endocrine**	12	Papillary thyroid carcinoma	36 y	M	No *EXT1/EXT2* alterations
	13	MEN Type 1	47 y	M	*EXT1* and MEN1 mutations

**Abbreviations:** AML: Acute Myeloid Leukemia; ALL: Acute Lymphoblastic Leukemia; NHL: Non-Hodgkin Lymphoma; BL: Burkitt Lymphoma; SCC: Squamous Cell Carcinoma; MEN: Multiple Endocrine Neoplasia.

## Data Availability

No new data were created or analyzed in this study. Data sharing is not applicable to this article.
